# Maxillary osteosarcoma in a beef suckler cow

**DOI:** 10.1186/2046-0481-65-15

**Published:** 2012-07-12

**Affiliations:** 1 Scottish Centre for Production Animal Health & Food Safety, School of Veterinary Medicine, University of Glasgow, Bearsden Road, Bearsden, G61 1QH, UK; 2 Paragon Veterinary Group, Townhead Farm, New Biggin near Stainton, Penrith, CA11 0HT, UK; 3 Vetmeduni Vienna, Clinic for Ruminants, Veterinärplatz 1, A 1210, Vienna, Austria; 4 School of Veterinary Science, University of Bristol, Langford House, Langford, Bristol, BS40 5DU, UK

**Keywords:** Osteosarcoma, Cow, Neoplasia, Maxilla

## Abstract

A ten-year-old beef suckler cow was referred to the Scottish Centre for Production Animal Health & Food Safety of the University of Glasgow, because of facial swelling in the region of the right maxilla. The facial swelling was first noticed three months earlier and was caused by a slow growing oral mass which contained displaced, loosely embedded teeth. The radiographic, laboratory and clinicopathological findings are described. Necropsy, gross pathology and histological findings confirmed the mass as a maxillary osteosarcoma.

## Background

Facial swelling in farm animals can be caused by various diseases and trauma and often involve soft tissues or bone of the oropharynx. Well known causes of facial swelling in cattle are actinomycosis and actinobacillosis, but soft tissue abscesses, dental or periodontal diseases, facial bone fractures, salivary gland or sinus problems and neoplasia can all be underlying causes of facial swelling. Abattoir surveys indicate that the general incidence of neoplasms of the oropharynx in cattle is low [1,2] with tumours such as carcinomas, fibromas, sarcomas and papillomas [3-6] reported most commonly. These tumours can occur spontaneously or after chronic ingestion of foodstuff with a mutagenic effect such as bracken fern [2,6,7].

Correct classification of tumours of the oropharynx can be difficult as a considerable interrelationship among epithelial structures, soft mesenchymal structures, bone and cartilage can result in tumours of mixed origin [4,8]. Diagnostic imaging, pathology and other diagnostic methods can be used to correctly diagnose oral masses, consider possible treatment options and predict prognosis. This case report describes the radiographic, laboratory and clinicopathological findings of a rare maxillary osteosarcoma in a beef suckler cow.

## Case report

**Figure 1:**
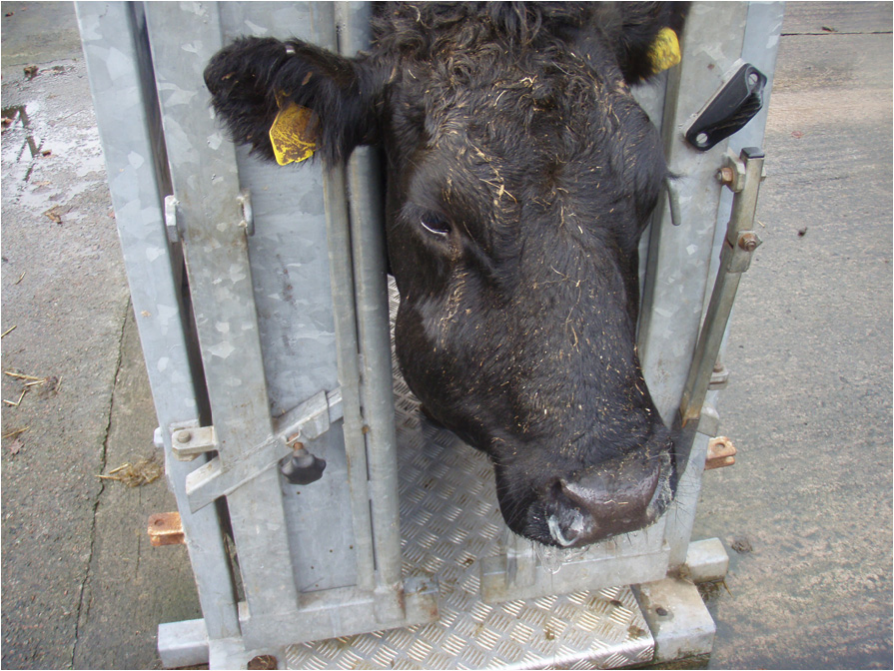
Unilateral facial swelling in a 10-year-old Limousin cross cow.

A ten-year-old Limousin cross cow was referred to the Scottish Centre for Production Animal Health & Food Safety of the University of Glasgow with facial swelling of the right maxilla (Figure 1). The swelling was first noticed approximately three months earlier and had continued to enlarge since then. Approximately two weeks before referral the cow started to loose body condition and developed diarrhoea. It was assumed that the swelling interfered with proper mastication and the referring veterinarian referred the animal assuming soft tissue swelling in the oral cavity caused by a tooth root infection. No treatment was given at this stage.

**Figure 2:**
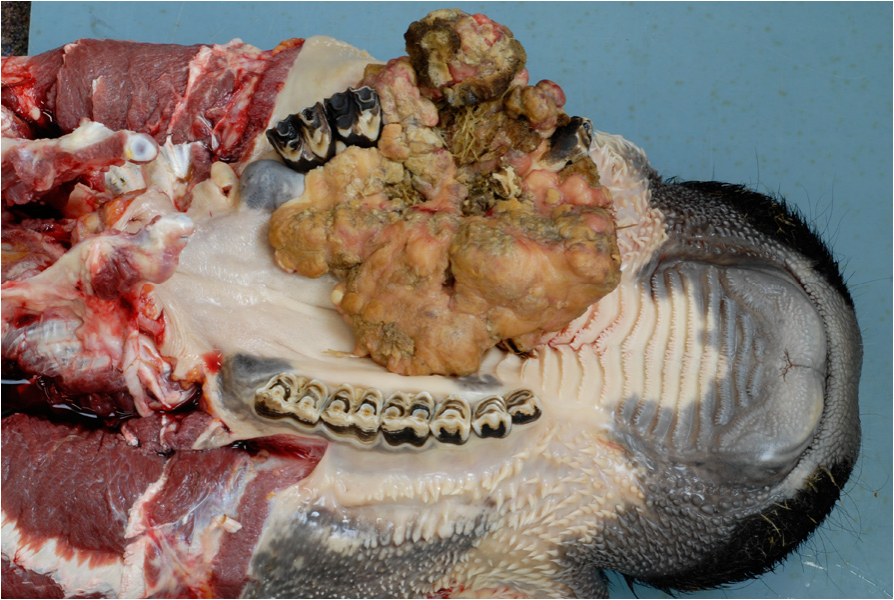
Oral mass protruding from the right maxilla.

Upon arrival, the cow was bright, alert and showed normal appetite and mastication. However, profuse diarrhoea was present. Unilateral facial swelling was noticed (Figure 1). The body condition score was 2 out of 5 [9]. On physical examination the cow had a normal rectal temperature (38.3°C) and respiratory rate (24 breaths/minute) but was tachycardic (94 bpm). A large firm swelling (approximately 25 × 15 cm) was present on the right maxilla. Air movement from both nares was detected and a bilateral mucopurulent nasal discharge was present. On oral inspection, a firm, ulcerated, non-painful mass, protruded from the right maxilla (Figure 2). Halitosis was marked. The mass contained displaced, loosely embedded maxillary teeth.

Heamatology results revealed a leukopenia (2.84 × 10^9^/L) and lymphocytopenia (1.193 × 10^9^ /L). Biochemical analysis indicated low calcium (1.94 mmol/L), magnesium (0.48 mmol/L) and albumin (16 g/L) concentrations while globulin (72 g/L) and total protein (88 g/L) concentrations were slightly elevated; the albumin/globulin ratio was decreased. No abnormal findings were reported on bacteriology and parasitology of a faecal sample. Bovine virus diarrhoea (BVD) antibody detection was positive but virus detection was negative. Johne’s disease serology was negative.

Culturing of samples taken from the mucopurulent nasal discharge isolated the following bacteria and fungi: *Trueperella (Arcanobacterium pyogenes), Pseudomonas aeruginosa, Staphylococcus epidermidis, Streptococcus spp*., and *Candida spp*. A small superficial section (1 × 1 cm) of the mass was removed for bacteriological and histopathological examination. Culture results of the mass indicated sparse cultures of *Streptococcus spp*., *Staphylococcus epidermidis* and *Weeksiella virosa*. Histological results showed infiltration of fibrous tissue with inflammatory cells, many of which contained bacteria. No signs resembling *Actinomyces bovis* were found, sulphur granules were not observed. Neoplastic cells were also not observed in this initial tissue sample.

Laterolateral radiographs of the head showed that the maxillary premolar and molar teeth were displaced or missing and that parts of the sinuses and oral cavity had been replaced by soft-tissue and bone opacities. Multiple gas-fluid interfaces were observed within the mass, suggestive of abscesses or necrotic tissue. Slight radiolucencies were observed in the maxilla. Although many maxillary premolar and molar teeth were displaced or missing, no radiographic signs suggestive of tooth root infection were present. A dorsoventral radiograph showed the three dimensional extent of the mass. The combination of bacterial, culture, histology and radiographic results failed to support a diagnosis of either, actinomycosis, actinobacillosis, tooth root infection, salivary gland or sinus pathologies or fracture of facial bones. Despite the absence of neoplastic cells in a sample of the mass, a presumptive diagnosis of neoplasia with secondary soft-tissue infection and bacterial osteomyelitis was made. A grave prognosis was given, but as the animal showed no clinical signs of pain it was decided to initiate treatment. An antimicrobial and non-steroidal treatment was initiated with clavulanic acid and amoxicillin (Synulox^tm^, Pfizer Ltd, Sandwich, Kent) and ketoprofen (Ketofen^TM^ 10%, Merial Animal Health Ltd, Harlow, Essex). Despite treatment, facial swelling continued to enlarge. Needle aspiration of the external buccal swelling confirmed the presence of purulent material. The external buccal skin was lanced and approximately 100 mL of purulent material was retrieved. Antimicrobial treatment was changed to procaine penicillin and dihydrostreptomycin sulphate (Pen & Strep, Norbrook®, Carlisle, Cumbria) following antibiotic sensitivity results of the cultured bacteria. However, soon there-after, mastication was severely impaired and the cow started to drop her cud and stopped eating. Given the perceived extent of the mass and the grave prognosis it was decided to euthanase the cow at this stage**.**

At necropsy the cow was thin and there was a bolus of food lodged in an outpouching of the right upper cheek. The cheek papillae were smooth. A firm, large oral mass was apparent (Figure 2). The mass was diamond shaped with its centre in the hard palate and the rostral point at the 8^th^ palatine ridge. The mass extended caudally for a distance of 18 cm to a point on the right palate opposite the last molar tooth. The lateral point of the mass lay beyond the right upper dental arcade where the growth of the mass had either displaced or destroyed the molar teeth. The surface of the mass was nodular and irregular and was composed of firm fleshy grey-white tissue to which food was adherent. The centre of the mass formed open units, lined with foul smelling tissue above which the same grey-white tissue could be found. The mass filled the palatine sinus and extended into the maxillary sinus. The texture of the mass in these areas was gritty. The mandibular lymph nodes were irregularly shaped and enlarged to approximately two times normal size. They contained multiple gritty foci. Poorly masticated food was present in the rumen but there was no evidence of tumour spread to other organs such as the lungs, despite an extensive post mortem examination. No other abnormalities were detected in the digestive tract.

**Figure 3:**
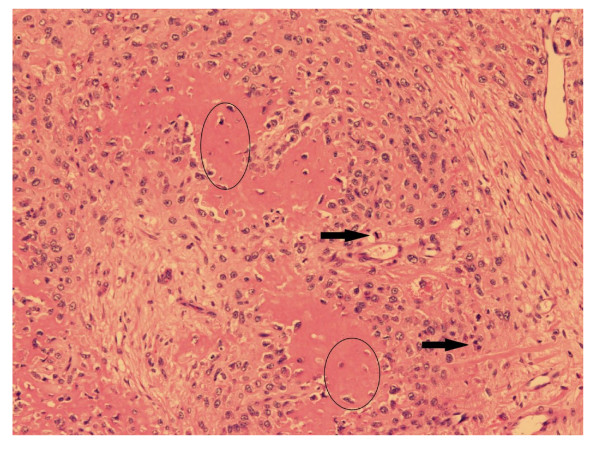
**Microscopic image of necropsy findings.** HE. *Osteosarcoma tumour with pleomorphic and spindle shaped tumour cells (white arrows) accompanied by osteoid (circles). Few mitotic figures can be seen (black arrows).*

Microscopic examination confirmed the mass as an osteosarcoma. The tumour cells were pleomorphic and spindle shaped with plump oval nuclei (Figure 3). In the superficial parts of the tumour, these cells were accompanied by fine strands of osteoid which became distinct islands, mineralized in places, in the deeper parts of the tumour. Mitotic figures were very common in the cellular areas (Figure 3).

There was a lymphadenitis composed of caseous type material in the mandibular lymph nodes. Sheets of macrophages distorted the node structure and there were areas of necrosis and calcification. Multinucleated giant cells were obvious. Acid fast organisms were not detected on smears performed of the node nor were they visible on histological sections. Rod shaped organisms were not detected on Gram stains performed of the node but a *Pasteurella*-like organism was isolated on routine bacteriological culture.

## Discussion

Clinical examination of this case revealed a profuse diarrhoea and a large oral mass causing facial swelling whilst necropsy also revealed lymphadenitis in the mandibular lymph nodes. No obvious reason for the diarrhoea was detected and it was hypothesized that the mass severely interfered with proper mastication and rumination causing a digestive upset resulting in diarrhoea. Although caseous lymphadenitis is a distinct disease entity of *Corynebacterium pseudotuberculosis*, Gram stains of the lymph node failed to identify *C. pseudotuberculosis.* The exact cause of the lymphadenitis in this case remains unclear, although it is assumed to be secondary to the tumour and its associated infection.

In this case, histological investigation undertaken post mortem revealed a diagnosis of maxillary osteosarcoma. These tumours are not frequently diagnosed in cattle in field circumstances, but if osteosarcomas are present they frequently develop in the skull of veterinary species [10-15] often causing facial swelling or deformities. Osteosarcomas are composed of tumour cells that produce osteoid or bone and typically result in bone destruction and new bone formation, invade adjacent soft tissues and often metastasize [12,16-18]. However this case and a previously described bovine osteosarcoma case [14] report invasion of adjacent tissues but absence of metastasis, suggesting that, although the tumour is locally very aggressive, it does not easily spread to other parts of the body of cattle. However, more reported cases of bovine osteosarcomas are needed to confirm this observation.

Osteosarcoma in humans and small animals are often treated with aggressive surgical procedures frequently in combination with radiation and/or chemotherapy [19]. In cattle these techniques are usually impractical [14] because of the location of the tumour and the hazardous consequences regarding food safety. In most cases any kind of treatment in cattle would not be economically feasible.

## Conclusions

Although several diagnostic methods were used, only after post mortem examination and histopathology was a definitive diagnosis of osteosarcoma made. The infiltrative nature, central areas of cavitation and necrosis, and the scattered areas of osteoid matrix are characteristic of osteosarcoma. Despite few reports in cattle, osteosarcoma should be included in the differential diagnosis of firm oral masses in cattle.

### Ethical approval

This individual clinical case was not involved in any experimental research and was thus not subject to prior review by an ethics committee, although its acquisition, diagnosis, treatment and subsequent slaughter falls within the approved ethical procedures of the University of Glasgow School of Veterinary Medicine.

## Competing interests

The authors declare that they have no competing interests.

## Authors’ contributions

Diether Prins drafted the work, performed the clinical work-up, sample collection, radiography and treatment. David Barrett supervised the clinical work regarding this case. Both Thomas Wittek and David Barrett were involved in the revision of the manuscript. All authors read and approved the final manuscript.
